# Current state of signaling pathways associated with the pathogenesis of idiopathic pulmonary fibrosis

**DOI:** 10.1186/s12931-024-02878-z

**Published:** 2024-06-17

**Authors:** Yang Zhou, Tingting Ling, Weihong Shi

**Affiliations:** School of Medicine, Jiangsu Vocational College of Medicine, Yancheng, Jiangsu 224005 China

**Keywords:** Idiopathic pulmonary fibrosis, Signaling pathways, Review

## Abstract

Idiopathic Pulmonary Fibrosis (IPF) represents a chronic and progressive pulmonary disorder distinguished by a notable mortality rate. Despite the elusive nature of the pathogenic mechanisms, several signaling pathways have been elucidated for their pivotal roles in the progression of this ailment. This manuscript aims to comprehensively review the existing literature on the signaling pathways linked to the pathogenesis of IPF, both within national and international contexts. The objective is to enhance the comprehension of the pathogenic mechanisms underlying IPF and offer a scholarly foundation for the advancement of more efficacious therapeutic strategies, thereby fostering research and clinical practices within this domain.

The clinical practice guideline “idiopathic pulmonary fibrosis an update and progressive pulmonary fibrosisin adults An official ATS ERS JRS ALAT(2022)” provided a unified approach to the process of progressive worsening of interstitial lung disease (ILD) other than idiopathic pulmonary fibrosis (IPF). It introduced the term “progressive pulmonary fibrosis” (PPF) for the first time to describe and specify diagnostic criteria, replacing progressive fibrosing ILD (PF-ILD), and provided treatment recommendations [1]. IPF is characterized by irreversible fibrosis of unknown etiology, exclusive to adults, confined to the pulmonary domain, and marked by progressive fibrosis in interstitial pneumonia. IPF carries a grim prognosis, with an estimated mean survival of 2–5 years from diagnosis. Mortality rates are significant, with 64.3 deaths per million in men and 58.4 deaths per million in women [2]. Moreover, mortality rates tend to rise with advancing age, consistently higher in men compared to women. Interestingly, there is a seasonal pattern to mortality, with peak death rates occurring in winter, even after excluding infectious causes. The pathogenesis of IPF is intricately shaped by the interplay between genetic factors and environmental risks, although the precise causes and molecular mechanisms remain incompletely elucidated [3]. Diverse signaling pathways significantly contribute to the onset and progression of IPF, impacting signal transduction and modulating lung function. A strategic exploration of the roles and interactions among these signaling pathways in the pathogenesis of IPF, coupled with targeted interventions to impede disease progression, holds promise as an effective avenue for developing treatments for IPF.

## Transforming growth factor-β (TGF-β)/smads signaling pathway

TGF-β, a multifunctional cell cytokine classified within the transforming growth factor superfamily, boasts over 40 members identified since the discovery of its fibroblast trans differentiation capabilities in 1981 [4]. In mammals, these cytokines govern cell growth, differentiation, migration, apoptosis, and extracellular matrix (ECM) production. TGF-β manifests in three isoforms: TGF-β1, TGF-β2, and TGF-β3, all activated in the progression of various fibrotic diseases [5]. Particularly, TGF-β1 emerges as a pivotal mediator in fibrosis and inflammation, with its receptors, TGF-βRI and TGF-βRII, assuming crucial roles in epithelial-mesenchymal transition (EMT) and fibrosis formation [6]. Downstream molecules encompass Smad2, Smad3, Smad4, and Smad7. While TGF-β1 activates the downstream effectors Smad2 and Smad3, it encounters negative regulation from Smad7 [7]. Consequently, under fibrotic conditions, there is an upregulation of Smad2 and Smad3 expression, coupled with downregulated Smad7. Initially secreted in an inactive form into the ECM, TGF-β1 forms a complex with latency-associated peptide for storage [8]. Upon activation by diverse cellular signals, TGF-β1 transforms into a dimer connected by disulfide bonds. This dimer binds to TGF-βRII, inducing the phosphorylation of TGF-βRII and subsequently activating TGF-βRI kinase. Activated TGF-βRI phosphorylates cytoplasmic effectors Smad2 and/or Smad3, forming a heterotrimeric complex with Smad4. This complex translocates into the nucleus, binds to common sequences, and directly or indirectly regulates gene transcription [9]. This intricate signaling cascade instigates a series of intracellular signals, ultimately promoting the transcription of nuclear factors Snail and Twist, inhibiting the production of endothelial markers, and activating the expression of mesenchymal markers [10]. TGF-β activation culminates in the excessive production of ECM components, including fibrinogen and fibronectin, which intricately control TGF-β activation either directly or indirectly.

The TGF-β/Smad signaling pathway is a multifunctional cascade pivotal in inflammation, wound healing, and fibrosis, significantly contributing to ECM deposition and EMT during tissue fibrosis [11]. Using human lung fibroblasts WI-38, Kono et al. explored the rationale of sphingosine kinase (SPHK) activation with TGF-beta1 stimulation, which leads to Rho-associated myofibroblast differentiation mediated by transactivated S1P receptors in the lung fibrogenic process [12]. TGF-β1 exerts inhibitory effects on Smad3/4 by inducing the overexpression of Smad7. Functioning as an inhibitor, Smad7 suppresses ubiquitination of Smad2 and Smad3, inhibiting SMURF2 ubiquitin ligase and consequently preventing phosphorylation This process facilitates the degradation of the TGF-βRI and RII complex, ultimately impeding TGF-β1 signaling. TGF-β induces C/EBPβ acetylation in human alveolar epithelial cells, resulting in increased α-SMA expression [13]. Additionally, TGF-β engages in a complex molecular interaction network, promoting EndMT and interactions with other signaling pathways [14]. Elevated expression of the heat shock protein HSP90 in IPF patients identifies it as a potential biomarker and therapeutic target for lung fibrosis [15]. Recent studies reveal that inhibiting HSP90 effectively blocks the TGF-β/Smad signaling pathway. In IPF patients, heightened TGF-β1 levels in airway epithelium and fibroblasts lead to fibroblast differentiation and aberrant injury responses, exacerbating progressive fibrosis. In mouse models, inhibition of the TGF-β signal delays lung fibrosis progression [16]. Alveolar macrophages, significant sources of TGF-β1, induce EMT when co-cultured with mouse lung epithelial cells. The TGF-β receptor inhibitor LY2109761, pre-treated with macrophages, blocks EMT, demonstrating that alveolar M2 macrophages induce EMT through the TGF-β1/Smad signaling pathway [17]. In transgenic mouse models overexpressing TGF-β1, inhibiting the accumulation of alveolar macrophages suppresses bleomycin-induced lung fibrosis [18]. Although therapeutic approaches targeting molecules in the TGF-β signaling pathway alleviate lung fibrosis in model mice, these studies remain experimental and have not progressed to clinical research. Inhibiting TGF-β activity, without disrupting its normal physiological functions, may offer a novel approach to fibrosis treatment by targeting downstream signaling effectors mediating fibrosis.

## Mitogen-activated protein kinase (MAPK) signaling pathway

MAPK, a group of serine-threonine protein kinases, responds to various stimuli, engaging in the intracellular transduction of multiple signals. In mammals, the MAPK family comprises three major phosphorylated kinases: Extracellular Regulated Protein Kinases (ERK), p38MAPK, and c-Jun N-terminal kinases (JNK) [19]. External factors can directly activate MAPK family proteins, initiating signal transduction into the cell nucleus, thereby participating in gene transcription, cell regulation, and tissue fibrosis associated with inflammatory reactions, as well as various physiological and pathological processes.

ERK signal transduction follows a three-tiered enzymatic cascade reaction within the MAPKs. ERK1 and ERK2, the most extensively studied members of the ERK family, partake in diverse signal pathways governing cell growth, proliferation, and differentiation [20]. Elevated expression of both ERK1 and ERK2 in lung tissues of bleomycin-induced IPF rats implies the mediation of the pathological process of IPF by the ERK signaling pathway [21]. Disruption of the ERK pathway allows ex vivo-cultured primary human mesothelial cells from peritoneal dialysis effluent to regain an epithelial-like morphology, downregulating Snail1 expression, and preventing EMT. Increased levels of α-SMA and phosphorylated ERK protein in IPF rat lungs, accompanied by elevated Rasp21 expression levels, suggest a collaborative role between the Smad and ERK pathways in collagen synthesis under the induction of TGF receptors [22].

p38MAPK, constituting a classic pathway in the MAPK signaling cascade, involves a four-kinase cascade: PAK (p21-activated kinase, MAPKKKK), MLK (MAPKKK, MKKK, or MEKK), MKK3/6/4 (MAPKK, MKK, or MEK), and p38MAPK (MAPK). Activation of p38MAPK promotes the expression and release of pro-inflammatory cytokines, disrupting inflammatory regulation balance and triggering a cascade reaction [23]. TGF-β1 induces p38 protein phosphorylation through receptors, activating the p38MAPK signaling pathway and stimulating collagen synthesis by lung fibroblasts. The interaction between p38MAPK and the TGF-β signaling pathway contributes to IPF formation and development. The p38MAPK inhibitor SB203580 exhibits anti-IPF effects by inhibiting TGF-β expression and p38MAPK phosphorylation, thereby suppressing EMT progression [24].

JNK, primarily residing in the cytoplasm, swiftly translocates to the cell nucleus upon activation, influencing the expression of corresponding genes. JNK’s activity modulates the IPF process by altering TGF-β1 activity. In the bleomycin-induced IPF model in C57BL/6 mice, mutual expression and interaction of JNK and Smad signaling pathways play a crucial role in IPF progression [25]. Activated JNK during IPF, accompanied by increased type I collagen secretion, can be significantly inhibited by the JNK-specific inhibitor SP600125, resulting in decreased type I collagen secretion and significant inhibition of extracellular matrix (ECM) processes. Application of the MAPK pathway inhibitor PD98059 in bleomycin-induced IPF mice attenuates ERK phosphorylation and inflammatory factor phosphorylation of JNK protein, alleviating lung inflammatory reactions and interstitial fibrosis, thereby providing clear protective effects against bleomycin-induced lung injury [26]. Xylourgidis et al. demonstrated in a bleomycin-induced pulmonary fibrosis model that MKP-5-deficient mice showed protection against lung fibrosis development. They exhibited decreased levels of hydroxyproline and fibrogenic genes, along with a notable shift towards an M1-macrophage phenotype. Our study revealed that the profibrogenic effects of TGF-β1 were suppressed in MKP-5-deficient lung fibroblasts. These fibroblasts displayed heightened p38 MAPK activity, impaired Smad3 phosphorylation, elevated Smad7 levels, and reduced expression of fibrogenic genes. Furthermore, myofibroblast differentiation was attenuated in MKP-5-deficient fibroblasts [27].

## Wnt/β-catenin signaling pathway

Wnt comprises 19 secreted glycoproteins primarily involved in regulating mammalian embryonic development and tissue repair [28]. The Wnt signaling pathway encompasses the classical Wnt/β-catenin pathway, the non-classical planar cell polarity pathway, and the non-classical Wnt/calcium pathway. Upon binding to the Frizzled receptor and the co-receptor low-density lipoprotein receptor-related protein on the cell surface, Wnt activates the Wnt/β-catenin pathway. This activation leads to the stimulation of Dishevelled (DSH) receptor family proteins, resulting in alterations in nuclear β-catenin levels. DSH, a crucial component of the cell membrane-associated Wnt receptor complex, inhibits downstream protein complexes, including Axin, GSK-3, and APC proteins [29]. The Axin/GSK-3/APC complex promotes the degradation of intracellular signaling molecule β-catenin. When the degradation complex is inhibited, stable β-catenin accumulates in the cytoplasm, with a portion entering the nucleus to interact with TCF/LEF transcription factor families and facilitate the expression of specific genes, including MMP genes, cell cycle regulatory factor genes, and oncogenes [30].

Various Wnt proteins, such as Wnt2, Wnt3A, Wnt5A, Wnt5B, Wnt7B, Wnt10A, Wnt11, and Wnt13, are detected in human lung tissue. Wnt/β-catenin signaling significantly contributes to pathological processes in the lungs, including inflammation, remodeling, and fibrosis [31]. Lung biopsy results from IPF patients indicate that the highly activated classical Wnt/β-catenin signaling pathway correlates with tissue repair and fibroblast activation. Research also suggests that Wnt/β-catenin signaling participates in inducing EMT during fibrosis development. Expression of Wnt1, Wnt3A, Wnt7B, Wnt10B, Fzd2, Fzd3, and β-catenin notably increases in IPF patient lung tissues [32]. Ligands Wnt5A and Wnt5B influence lung fibroblast differentiation through TGF-β, with many studies positioning the Wnt signaling pathway downstream of the TGF-β signal. Elevated Wnt/β-catenin signaling levels promote fibroblast migration and proliferation, suggesting its common involvement in lung fibrosis [33]. However, literature reports indicate that the Wnt signal may act upstream of TGF-β, with upregulated Wnt signal transduction leading to overexpressed TGF-β, inducing EMT and regulating tissue repair, causing the accumulation of extracellular matrix (ECM), such as collagen and MMP [34]. Aberrant Wnt signaling indicates the occurrence and development of IPF, contributing to EMT, ECM deposition, lung fibroblast proliferation, and myofibroblast differentiation. Downregulating the Wnt signaling pathway inhibits myofibroblast differentiation, thereby ameliorating pulmonary fibrotic lesions. In animal models of IPF, the Wnt/β-catenin signaling pathway is significantly activated, and blocking it attenuates lung fibrosis in mice [35]. Moreover, the Wnt/β-catenin signaling pathway interacts with other pathways involved in IPF progression.

## PI3K/AKT signaling pathway

The PI3K family of proteins plays a regulatory role in multiple signal transduction pathways, actively contributing to the process of tissue fibrosis. Upon activation, PI3K generates the second messenger PIP3 on the cell membrane, subsequently activating AKT downstream of PI3K through binding to PIP3 [36]. Studies have elucidated that Angiotensin II (AngII) modulates fibroblast proliferation, AKT phosphorylation, and PI3K activity via receptors AKT1 and AKT2, consequently activating the PI3K/AKT pathway [37]. This suggests that AngII potentially influences fibroblast biological behavior through mediating the PI3K/AKT pathway. In a bleomycin-induced mouse model of IPF, the PI3K/AKT/HIF-1α signaling pathway exhibits significant activation in lung tissue [38]. Notably, surfactant protein ProSPC production diminishes, collagen III content and mRNA levels increase, and there is a substantial rise in apoptotic cell numbers in the lungs [39]. This implies that abnormal activation of the PI3K/Akt/HIF-1α signaling pathway contributes to excessive apoptosis and abnormal cell proliferation, thereby fostering IPF development. During TGF-β1-induced EMT in human peritoneal mesothelial cells, inhibiting PI3K/AKT leads to reduced SMURF2 expression and an elevation in Smad2 protein expression, indicating a positive correlation between SMURF2 expression and PI3K/AKT, and a negative correlation between SMURF2 expression and Smad2 [40]. TGF-β1 not only induces EMT through Smad but also activates the PI3K/AKT signaling pathway, resulting in increased SMURF2 protein expression, enhancing Smad2 degradation, and further promoting the EMT process. In summary, the PI3K/AKT signaling pathway plays a positive regulatory role in the mesenchymal transition of peritoneal epithelial cells induced by TGF-β1. Tetrandrine (TET), derived from Stephania tetrandra S. Moor, is a bisbenzylisoquinoline alkaloid known for its potential therapeutic effects in attenuating the progression of silicosis. Ruimin et al. found through Molecular Docking analysis that TET binds to AKT1, the catalytic subunit of PI3K, and KDR. TET significantly alleviates silica-induced pulmonary fibrosis and reduces the expression of fibrotic markers In vivo. These findings suggest that TET has the potential to suppress silica-induced pulmonary fibrosis by targeting the PI3K/AKT signaling pathway, providing valuable insights into its therapeutic potential for the treatment of pulmonary fibrosis and silicosis.

## Rho/Rock signaling pathway

The Rho protein family constitutes a guanosine triphosphate (GTP)-binding group, featuring around 20 members in the RhoGTPase superfamily, with Rho, Cdc42, and Rac being the most extensively studied. Rho-associated kinase (Rock), a serine/threonine protein kinase and a crucial downstream effector of Rho, comprises two subtypes, Rock1 (p160Rock) and Rock2 (Rho kinase), with Rock1 being the predominant subtype expressed in the lungs [41]. The downstream myosin light chain (MLC) in the Rho/Rock signaling pathway serves as a significant contributor to cell migration force on the extracellular matrix (ECM), facilitating cell skeleton reorganization and engaging in biological behaviors such as phagocytosis, migration, contraction, and adhesion [42]. In vitro establishment of a low-oxygen microenvironment for human embryonic lung fibroblasts results in aberrant expression of connective tissue growth factors, contributing to IPF development. In a bleomycin-induced mouse model of IPF, heightened Rock1 and α-SMA expression alongside decreased E-cad expression suggest the involvement of the Rho/Rock signaling pathway in the epithelial-mesenchymal transition (EMT) process during IPF [43]. Research indicates that the Rho/Rock signaling pathway may also contribute to the IPF process by modulating the secretion of inflammatory mediators and the infiltration of inflammatory cells. In the context of TGF-β1-stimulated differentiation of lung fibroblasts into myofibroblasts, increased expression of α-SMA, p-RhoA, Rock, SPF, p-MBS, type I, and type III collagen indicates TGF-β1’s potential to activate the Rho/Rock signaling pathway in rats, stimulating lung fibroblast differentiation into myofibroblasts, promoting collagen synthesis, and expediting IPF formation [44]. In a bleomycin-induced IPF rat model, Y-27,632 demonstrates inhibitory effects on fibroblast migration through the Rho/Rock signaling pathway, thereby decelerating the progression of IPF.

## JAK/STAT signaling pathway

The JAK family, a subset of the non-receptor protein tyrosine kinase (PTK), encompasses four identified members: JAK1, JAK2, JAK3, and TYK2. Lacking the Src homology 2 (SH2) domain, JAKs cannot directly transduce extracellular signals [45]. STAT, a distinct protein family with the ability to bind to DNA, features a highly conserved SH2 domain that specifically binds to tyrosine-phosphorylated cytokine receptors. This mediates the interaction between JAKs and STAT, participating in the formation of STAT dimers [46]. The JAKs/STATs signaling pathway transmission involves cytokines binding to their receptors, leading to the clustering of JAK kinases linked to the receptor. Through reciprocal tyrosine phosphorylation, activated JAKs catalyze tyrosine phosphorylation on the receptor, creating docking sites for corresponding STAT proteins [47]. STAT proteins, with SH2 domains, are recruited to these docking sites, where they bind to the receptors and undergo phosphorylation [48]. Subsequently, STATs, in dimer form, enter the cell nucleus, bind to the promoter of target genes, and complete gene transcription and expression, participating in crucial biological processes such as cell proliferation, differentiation, apoptosis, and immune regulation [49]. In the bleomycin-induced rat model of IPF, aberrant expression of STAT1, excessive platelet-derived growth factor (PDGF) secretion, and increased plasminogen activator inhibitor-1 (PAI-1) expression positively correlate with collagen content, suggesting the potential involvement of the JAKs/STATs signaling pathway in the IPF process [50]. Activation of JAK2, STAT1, STAT3, and STATS pathways contributes to IPF development. The protective mechanism of IL-27 involves inhibiting the phosphorylation of STAT1 and STAT5, thereby suppressing JAK/STAT pathway activity and ultimately inhibiting IPF [51]. Another study observed increased expression of JAK, STAT1, and STAT3 in lung tissues of IPF rats, which were downregulated after sophocarpine intervention, indicating that JAK/STAT signaling pathway activation promotes IPF [52].

## mTOR signaling pathway

The expression of mTOR in the lungs of patients with idiopathic pulmonary fibrosis (IPF) exhibits a close correlation with fibrosis scores and diminished lung function, indicating its potential association with the prognosis of lung fibrosis [53]. Excessive activation of mTOR is observed in the bleomycin-induced mouse model of lung fibrosis, contributing to the formation of pulmonary fibrosis [54]. In the bleomycin-induced rat model of lung fibrosis, the protein expression levels of PI3K, p-Akt, and p-mTOR in lung tissues are significantly elevated compared to the normal control group [55]. Lentivirus-mediated mTOR-small interfering RNA (siRNA) effectively inhibits mTOR expression in lung tissues of rats poisoned by paraquat, alleviating lung tissue damage and fibrosis [56]. The mTOR signaling pathway also participates in the fibrosis repair process of high oxygen lung injury. Rapamycin and mTOR-siRNA can inhibit the mTOR signaling pathway, protecting premature rats from lung injury caused by high oxygen and having a certain inhibitory effect on lung fibrosis. The overactivation of mTOR in alveolar epithelial cells and impaired lung autophagy are related to the pathogenesis of lung fibrosis [57]. The modulation of mTOR, either inhibition or enhancement, can be utilized for the treatment of lung fibrosis. Following lung injury, damaged alveolar epithelial cells release various chemotactic factors, recruiting mononuclear cells and neutrophils to the injured site.

In IPF, repeated cell damage and the inability to quickly eliminate neutrophils and macrophages lead to continuous production of reactive oxygen species, further exacerbating the fibrosis cascade reaction [58]. Neutrophil infiltration into the bronchoalveolar lavage is considered an early predictor of death in IPF patients, with macrophages and neutrophils identified as fibrosis-promoting cell types in a mouse model of lung fibrosis [59]. In normal lung tissue, Smad signaling can stimulate fibroblast proliferation and collagen deposition, creating a hypoxic environment that further activates the mTOR signaling pathway, promoting fibroblast survival and fibrosis progression. TGF-β-mediated Akt signaling can further activate the mTOR signaling pathway, regulating the inflammatory response of cells such as monocytes and macrophages [60].

The epithelial sodium channel (ENaC) is a crucial component for clearing alveolar edema fluid. Research indicates that activating the PI3K/Akt/mTOR signaling pathway can positively regulate ENaC, inhibiting the upregulation of sodium-potassium ATP pumps in stimulated alveolar epithelial cells, clearing excess fluid in the lungs, and alleviating lung tissue damage [54]. In lung fibrosis, the mTOR signaling pathway is involved in the regulation of myofibroblast apoptosis. An abnormal PTEN/Akt/mTOR pathway inhibits autophagy, desensitizing IPF fibroblasts from stress induced by aggregated collagen, resulting in an anti-apoptotic phenotype and maintaining vitality on collagen [61]. Long non-coding RNA (lncRNA) can regulate adjacent genes, and the protein-encoding gene ribosomal protein S6 kinase β2 (RPS6KB2) is adjacent to lncRNA AP003419.16. Scholars believe that RPS6KB2 is involved in aging and IPF, and the activation of RPS6KB2 is regulated by the protein kinase mTOR signaling pathway [62]. Research indicates a significant increase in the expression of AP003419.16 in IPF patients, along with an increase in RPS6KB2, suggesting a correlation between aging and IPF. Further studies show that mTORC1 activation reduces autophagy in aging and IPF, and the activation of this pathway also helps resist fibroblast death in IPF.

## VEGF, FGF, PDGF signaling pathways

The VEGF family comprises VEGF-A, VEGF-B, VEGF-C, and VEGF-D [63]. The biological effects of VEGF hinge on its interaction with the tyrosine kinase receptors VEGFR1 and VEGFR2. VEGFR2 primarily transduces VEGF signaling in lung cells. Upon binding of free VEGF to cell surface VEGFR, it initiates phosphorylation and activation of downstream signaling pathways, notably the PI3K-Akt pathway and the focal adhesion kinase (FAK) pathway, contributing to fibrosis development [64]. Activated FAK enhances cell migration, infiltration, and resistance to apoptosis through integration with TGF-β. Inhibition of FAK can prevent experimental lung fibrosis and the formation of lung myofibroblasts. In the bleomycin-induced IPF model, FAK inhibitors or FAK silencing via siRNA significantly halt lung fibrosis [65]. The expression of VEGF significantly increases in the lung tissue of animals injected with bleomycin. The use of VEGFR antagonists can alleviate pathological fibrosis and collagen deposition in mouse lung fibrotic tissue. VEGF-A also exerts stimulatory effects on platelet-derived growth factor receptors (PDGFR), thus regulating the migration and proliferation of mesenchymal cells [66]. In the lungs of IPF patients, VEGF is mainly secreted by type II alveolar epithelial cells and lung fibroblasts.

The FGF family encompasses over 20 secreted proteins that regulate downstream signaling pathways, such as Ras/MAPK/ERK-1, PI3K/Akt, by binding to the tyrosine kinase-active transmembrane protein FGFR on the cell surface, thereby modulating cell proliferation, activation, and migration [67]. Some FGF members play a pro-fibrotic role by promoting the mitotic activity of fibroblasts, while others exhibit anti-fibrotic effects by fostering the regeneration and proliferation of epithelial cells. FGF-1 inhibits TGF-β1-stimulated myofibroblast differentiation and EMT, demonstrating an anti-fibrotic role [68]. FGF-10 sustains the clonal expansion and differentiation of alveolar epithelial progenitor cells, shielding them from oxidative stress, asbestos-induced DNA damage, and apoptosis [69]. In progressive IPF patients, FGF-10 expression is significantly reduced in alveolar interstitial stromal cells compared to stable IPF cases. FGF-9 and FGF-18 promote the survival and migration of human lung fibroblasts and inhibit the differentiation of myofibroblasts in vitro [70]. FGF-18 reduces myofibroblast differentiation, while FGF-7 and FGF-10 stimulate alveolar epithelial cell proliferation, reducing epithelial damage and apoptosis. Basic fibroblast growth factor (bFGF) or FGF-2 acts as a potent mitogen for fibroblasts, airway smooth muscle cells, and type II alveolar epithelial cells, inducing collagen synthesis in lung fibroblasts and myofibroblasts [71]. TGF-β upregulates FGF-2 expression, and FGF-2 synergistically enhances TGF-β1-induced fibroblast proliferation through the activation of the MAPK and PI3K-Akt pathways. Targeting the interstitial FGF signaling pathway can be considered a therapeutic strategy for IPF.

The PDGF family comprises PDGF-A, PDGF-B, PDGF-C, and PDGF-D, forming homodimers or heterodimers through disulfide bonds and presenting in five subtypes (AA, AB, BB, CC, and DD) [72]. PDGF dimers bind to and phosphorylate two receptor tyrosine kinases (PDGFR-α and PDGFR-β) in the form of homodimers or heterodimers with varying affinities. PDGFR-α and PDGFR-β, often derived from stromal cells in organs, induce receptor phosphorylation upon binding to PDGF, activating downstream signaling pathways, including MAPK and PI3K/Akt [73]. PDGF, a common growth factor produced in various cells, acts as a potent mitogen for fibroblasts, playing a crucial role in fibroblast proliferation. Excessive PDGF production can induce fibrosis in the heart, liver, and kidneys, with associations observed in various organs. PDGF promotes fibrosis through its mitogenic and chemotactic properties, and it can also enhance fibrosis by interfering with TGF-β, including the regulation of TGF-β levels. In lung tissues of IPF patients, the expression of PDGF increases in epithelial cells and alveolar macrophages [74]. Treating human lung fibroblasts with PDGF promotes the expression of collagen I and α-SMA by activating the MAPK pathway. Targeting PDGFR-β in animal models of IPF can alleviate bleomycin-induced IPF [75]. Therefore, PDGF is considered a crucial pathogenic factor in lung fibrosis. Nintedanib, a clinically approved anti-fibrotic drug, acts as a tyrosine kinase inhibitor to block the activity of PDGFR, as well as FGFR and VEGFR [76]. In IPF patients, nintedanib inhibits the differentiation of fibroblasts into myofibroblasts and the proliferation of myofibroblasts. However, the primary anti-fibrotic effects of nintedanib and its main target in fibrosis, particularly whether PDGFR is the principal target, remain uncertain.

## Conclusion

IPF represents a multifaceted ailment characterized by aberrations in numerous cytokines and signaling pathways throughout its inception and progression. While this manuscript delineates various IPF-related signaling pathways, numerous additional pathways assume pivotal roles in the pathogenesis of IPF. It is crucial to acknowledge that none of these signaling pathways operates autonomously; rather, there exists an inherent level of crosstalk and interaction, collectively orchestrating the regulation of IPF development. The continuous evolution of medical knowledge and the burgeoning repository of biological data are progressively enhancing our comprehensive understanding of signaling pathways associated with IPF. Delving deeply into the IPF signaling pathways facilitates a more profound exploration of genes and their functionalities linked to the disease. Moreover, it enables the identification of treatment targets and novel biomarkers crucial for early diagnosis.

## Data Availability

Dataset is available through request to the corresponding author.
